# Facilitating heart transplantability in an end-stage heart failure patient with brain abscess and infected left ventricle assist device—A unique case report

**DOI:** 10.1016/j.ijscr.2020.05.028

**Published:** 2020-05-22

**Authors:** Mohamad Ibrahim, Shawqi Arafat, Sebastian V. Rojas, René Schramm, Jan F. Gummert, Michiel Morshuis, Henrik Fox

**Affiliations:** Clinic for Thoracic and Cardiovascular Surgery and Heart Failure Department, Herz- und Diabeteszentrum NRW, Ruhr-Universität Bochum, Bad Oeynhausen, Germany

**Keywords:** Brain abscess, Left ventricular assist device, Heart transplantation, Antibiotic therapy, Case report

## Abstract

**Introduction:**

Heart transplantation is the desired therapy in end-stage heart failure (HF) and in patients with left-ventricular assist devices (LVAD) suffering from life-threatening complications on device. Comorbidities are frequent in end-stage HF and may refrain the patient from being listed for heart transplantation such as infections making surgery impossible.

**Presentation of case:**

We report the first case of a patient with proven LVAD system infection who additionally suffers from a brain abscess that made listing for heart transplantation impossible, leaving this patient no apparent treatment option.

**Conclusion:**

Our extraordinary treatment strategy finally resolved both the brain abscess and LVAD system infection through extensive conservative long-term treatment facilitating this patient to be finally listed for regular heart transplantation and sustained relieve of systemic infection.

## Highlights

• Therapeutic scope in a patient designated with no therapeutic option left.
• Strategy ineligible for heart transplantation for uncontrolled infection.
• Extraordinary case elucidating our unestablished treatment strategy.
• Finally after treatment patient was listed for heart transplantation.

## 1 Introduction

Heart transplantation (HTX) is the desired therapy in heart failure (HF) patients with left ventricular assist devices (LVAD) [1]. In particular, when it comes to LVAD therapy complications such as LVAD system infections, urgent HTX is required [2]. Nevertheless, there are various affections that can preclude HTX listing in these patients, such as infection, malignant tumors or psychiatric disorders [2]. Infectious diseases bear the potential of effective treatment, while severe infections like brain abscesses (BA) are challenging in identifying targeted therapy. BA is a life-threatening disease and most initial appearance is localized inflammation first, developing pus that encloses into a vascularized capsule [3]. Incidence of BA is estimated from 0.3 to 1.3 incidents per 100,000 individuals per year and the risk increases in immunocompromised, but also LVAD patients [3]. BA can derive from bacteria, fungus or parasites degenerating into systematic infectious and septic life-threatening conditions [3]. BA diagnosis can be challenging as symptoms are usually non-specific and frequently mirrored by the underlying disease [4]. Conductance of effective BA treatment permitting subsequent heart transplantation in LVAD patients is unknown and no recommendations of action in these cases are available. The spectrum of possible approaches ranges from conservative anti-infective treatment to surgical aspiration or complete surgical removal of the abscess [3], but the optimal approach to achieve heart transplantability in HF patients with infected LVAD and BA is entirely unknown. In this context we report a case from our academic center of a HF patient with infected LVAD and BA requiring urgent HTX and who was successfully treated for this BA with a conservative anti-infective regimen whereby surprisingly LVAD system infection is no longer traceable and the patient is now listed for HTX at our center. Our work is reported in line with the SCARE criteria [5].

## 2 Case report

We report of a 58-years old male, with medical history of end-stage HF for dilatative cardiomyopathy who has received a LVAD as bridge-to-transplant indication. The patient agreed with presentation of this unique case in this scientific journal. After recovery for LVAD implantation the patient complained about recurrent fever episodes, abnormal fatigue and elevated infection parameters as well as kidney retention measures in laboratory tests, but no focus of infection could be identified, and the patient received empiric intravenous antibiotic therapy with Ampicillin/Sulbactam. For persistence of the infection the antibiotic therapy was switched to Ciprofloxacin and later to Fosfomycin to extent the targeted germ spectrum, but without effect before the patient was transferred to our center. We identified the focus of infection and listing for HTX became urgently necessary with positron emission tomography (PET-CT) proving an anterior intrapericardial inflammation process that also embraced the outflow graft of the LVAD as well as caudal parts of the patient’s pacemaker lead.

Immediate HTX listing workup in our center revealed blood cultures positive for *Staphylococcus aureus* so we adapted antibiotic therapy for Flucloxacillin appropriately to the available antibiogram. In the course of this treatment the patient developed new episodes of fever (38.9 °C /102 °F) in addition to a mental state of confusion and the patient complained of visual constraints, including “dots and stars” in his field of vision. Neurological examination divulged left homonymous hemianopsia, but no other comprehensible pathology.

**Fig. 1:**
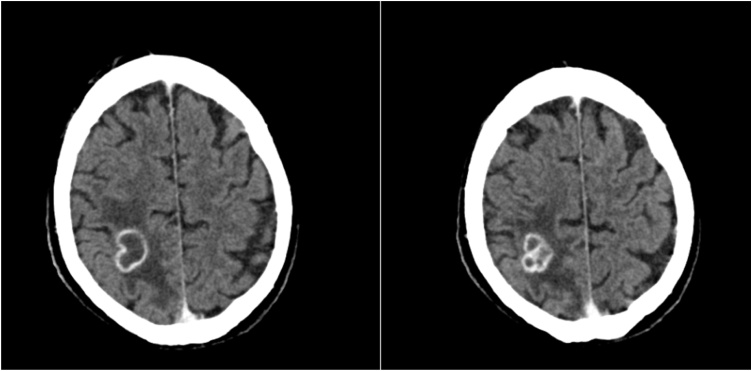
CT Scan with contrast with a 2.5 cm lesion at the right upper parietooccipital region.

Cerebral computed tomography (CCT) constituted spheres of atypical bleeding in both hemispheres and extra-axial blood formations in the left hemisphere with no dynamics in repeated CCT after six hours. Finally, contrast CCT found two hypointense parietal and occipital areas with scalloped microvascular blood impurities, with an hyperintense 2.5 cm ring enhancement with poorly definable circumvent at the right upper parietooccipital region including a surrounding edema (Fig. 1).

**Fig. 2:**
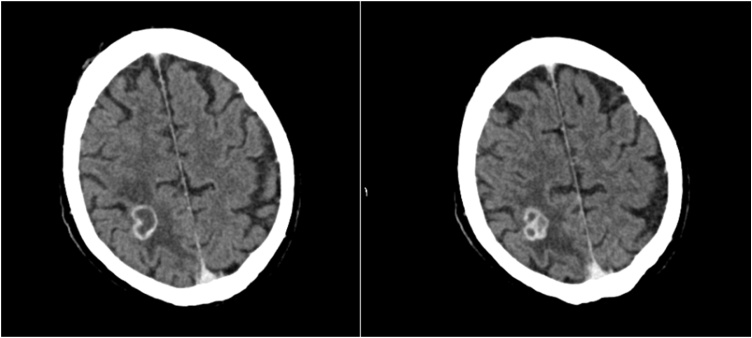
CT Scan with contrast with a 19 × 18 × 20 mm lesion and minimal regression of the surrounding edema.

This diagnosis of BA precluded the patient from prompt HTX, which was urgently indicated for the LVAD system infection. With antibiotic therapy of Rifampicin and Ceftriaxone the patient experienced relief of his visual symptoms as well as general condition and clinical presentation improved. Ceftriaxone was administered in dosage of 4 g daily to facilitate Ceftriaxone to pass hemato-encephalic barrier to reach BA. Contrast CCT after one week of treatment in our center showed slightly diminished lesions (19 × 18 × 20 mm) with an additional slight regression of the surrounding edema encouraging us to pursue the antibiotic regime to achieve HTX listing for this patient (Fig. 2).

**Fig. 3:**
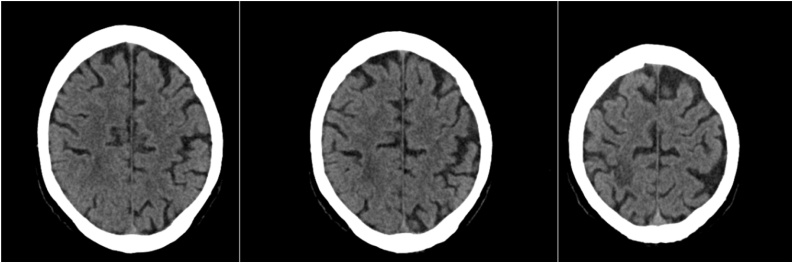
CT Scan with contrast a complete regression of the lesion and the surrounding edema.

Follow-up contrast CCT after seven weeks revealed marked BA and perifocal edema reduction. Antibiotic therapy was further continued to realize total resolution of the BA to enable HTX in this patient with PET-CT proven LVAD system infection and finally after thirteen weeks of antibiotic treatment CCT in conclusion publicized complete resolution of the BA (Fig. 3).

For optimal preparation for HTX the extent of the LVAD system infection was reassessed in PET-CT but surprisingly after thirteen weeks of broad-spectrum antibiotic therapy no LVAD system infection was traceable in PET-CT anymore and the patient has not experienced clinical significant infection recurrence afterwards.

## 3 Discussion

System infection of left ventricular assist device (LVAD) is considered a life-threatening condition and heart transplantation (HTX) is the best available treatment option [2,6]. However, various accompanying diseases such as infections can preclude patients from HTX. We report the first case of an end-stage heart failure patient with a LVAD system infection and simultaneous brain abscess (BA) in which both life-threating conditions were managed through a thirteen-week broadband antibiotic regime.

LVAD system infection is accepted as an indication for HTX, because no therapeutic approach, neither conservative nor surgical, is known or proven to resolve LVAD system infection [2]. Patients with BA are not eligible for HTX, demonstrating the dilemma of LVAD system infections, as they can promote BA in such patients through hematogenous spread of the germs even across hemato-encephalic barrier [7,8]. For the variable size and location of BA, BA are not represented by typical neurological symptoms such as headache, fever or alterations of consciousness making BA diagnosis often challenging [4]. Moreover, cerebral computed tomography (CCT), in particular when performed without contrast-dye, does not expedite clear and definite diagnosis of BA, especially in differentiating BA from other brain pathologies [9]. Magnet resonance imaging (MRI) is considered more sensitive and specific in BA diagnosis than CCT and helps to differentiate intracranial lesions, but MRI is prohibited in LVAD patients, as in our case [10].

Recommendations for BA treatment also vary with size and location of the BA in every individual patient, but in this context LVAD patients once more represent a peculiar cohort, because surgical interventions bear augmented bleeding risks in LVAD patients [11]. Moreover no recommendations for LVAD system infection are available and only few reports are published to report successful overcoming of LVAD system infection using approaches other than HTX [11], leaving those patients with LVAD system infection and contraindications for HTX in an irresolvable dilemma.

Furthermore, the microbial spectrum of BA and LVAD system infection is manifold, making the decision for targeted antibiotic therapy almost impractically. In addition to that 27% of BA are polymicrobial [3], while no reliable data for the germinal spectrum for LVAD system infection is available. *Staphylococcus aureus* as identified in our case is specifically disputing in treatment, and literature supporting effective BA or LVAD system infection treatment of *Staphylococcus aureus* is missing in total. The impact on LVAD system infection of antibiotic therapy metered for passing hemato-encephalic barrier aimed to treat BA is not known either [12].

In conclusion recommendations and literature for treatment of such patients as reported in this case is scarce or entirely missing, as clearly summarized in the systematic review by Arlotti et al. that addresses all controversial aspects of BA therapy management. They conclude that no recommendations for antibacterial treatment for BA treatment can be enunciated, for lack of sufficient evidence [13] in this field. Our case report provides the very first evidence of both successful BA, but also LVAD system infection treatment and finally offering this patient listing for HTX.

## 4 Conclusion

A patient with both brain abscess (BA) and system infection of left ventricular assist device (LVAD) at first instance appears untreatable, because BA prohibits life-saving heart transplantation. Our case is the first to describe a successful treatment strategy to finally resolve both BA and LVAD system infection, which has not been reported yet.

## Declaration of Competing Interest

All authors declare that there is no conflict of interest in context with this case report.

## Funding

Funding for this research was not obtained for this case report.

## Ethical approval

Case reports are exempt from ethical approval in our institution.

## Consent

Written informed consent was obtained from the patient.

## Author contribution

Mohamad Ibrahim MD: data collection, writing the paper.

Shawqi Arafat MD: data collection, writing the paper.

Sebastian V Rojas MD: data analysis and interpretation writing and reviewing the paper.

René Schramm MD PhD: data analysis and interpretation writing and reviewing the paper.

Jan F Gummert MD: data analysis and interpretation writing and reviewing the paper.

Michiel Morshuis MD: data interpretation writing and reviewing the paper.

Henrik Fox MD FHFA: study concept, study design, data analysis and interpretation, paper editing, writing and review.

## Registration of research studies

1. Name of the registry: N/A.
2. Unique identifying number or registration ID: N/A.
3. Hyperlink to your specific registration (must be publicly accessible and will be checked): N/A.

## Guarantor

Henrik Fox, MD FHFA.

## Provenance and peer review

Not commissioned, externally peer-reviewed.
